# Initial clinical presentation of single soft tissue metastasis of medullary thyroid carcinoma without primary tumor in the thyroid gland

**DOI:** 10.1186/s12957-017-1293-2

**Published:** 2017-12-13

**Authors:** Masanori Okamoto, Akira Takazawa, Kaoru Aoki, Yasuo Yoshimura, Hiroyuki Kato, Toshiaki Otsuki, Kazuma Maeno, Tomonobu Koizumi

**Affiliations:** 1 Department of Orthopaedic Surgery, Shinshu University School of Medicine, 3-1-1, Asahi, Matsumoto, Japan; 2 Department of Laboratory, Shinshu University School of Medicine, 3-1-1, Asahi, Matsumoto, Japan; 3 Division of Breast and Endocrine Surgery, Department of Surgery, Shinshu University School of Medicine, 3-1-1, Asahi, Matsumoto, Japan; 4 Department of Comprehensive Cancer Therapy, Shinshu University School of Medicine, 3-1-1, Asahi, Matsumoto Nagano, 390-8621 Japan

**Keywords:** Primary unknown origin, Calcitonin, TTF-1, Soft tissue metastasis, CEA

## Abstract

**Background:**

Single soft tissue metastasis of medullary thyroid carcinoma is extremely rare. In addition, several occult medullary thyroid carcinomas with distant metastasis were reported, but undetectable primary lesion at diagnosis was also extremely rare.

**Case presentation:**

A 74-year-old man was admitted to our hospital because of a painful nodule in his left buttock for over 1 year. Needle biopsy was performed, and the histological findings revealed adenocarcinoma positive for thyroid transcription factor-1. No evidence of a primary tumor, including the lung and thyroid gland, could be found elsewhere despite detailed examinations, including thyroid echography, chest computed tomography, and fluorodeoxyglucose-positron emission tomography. The soft tissue tumor was resected with a wide margin. Immunohistochemical analysis showed the tumor cells to be positive for cytokeratin-AE1/3, cytokeratin 7, synaptophysin, chromogranin A, calcitonin, and carcinoembryonic antigen, but negative for cytokeratin 20, Napsin A, Pax8, and p40, resulting in a diagnosis of metastasis of medullary thyroid carcinoma.

**Conclusion:**

Initial presentation with a single metastasis to soft tissue and undetectable primary tumor in the thyroid gland is an extremely rare clinical manifestation in patients with medullary thyroid carcinoma.

## Background

Medullary thyroid carcinoma (MTC) is an uncommon thyroid cancer accounting for 5–8% of thyroid neoplasms [1]. In contrast to common thyroid tumors, these tumors originate in calcitonin-producing C cells [1–3]. Regional metastasis to cervical lymph nodes occurs early in the disease, whereas MTC may spread hematogenously to affect the liver, lung, or bone [1–5].

We encountered a case of MTC with unusual clinical presentation as single soft tissue metastasis in the left buttock. The primary lesion in the thyroid gland was undetectable at diagnosis and after resection of the soft tissue metastasis. Here, we report the clinical course and discuss metastatic MTC.

## Case presentation

**Fig. 1:**
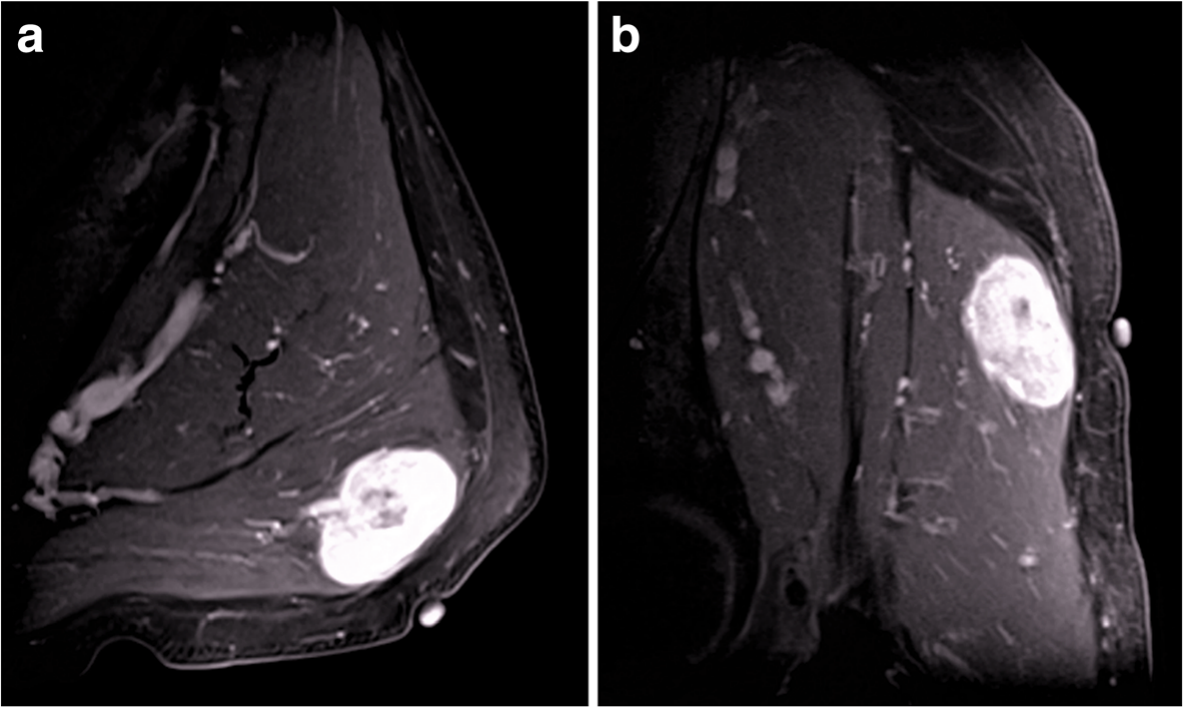
Magnetic resonance findings. **a** The axial T1-weighted image with intravenous gadolinium enhancement showed irregular peritumoral enhancement. **b** The sagittal enhanced T1-weighted image demonstrated a poorly enhanced region at the center of the tumor

**Fig. 2:**
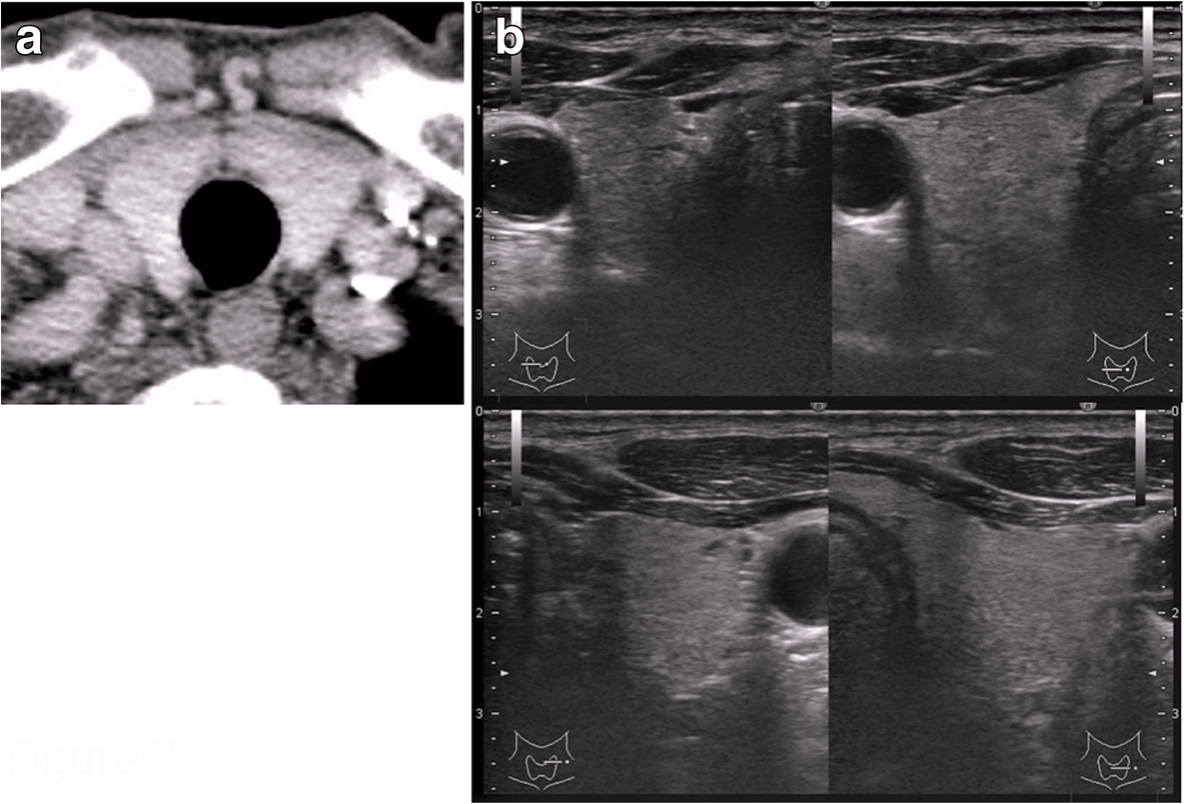
Computed tomography (**a**) and ultrasound (**b**) of the thyroid showed no abnormal findings

**Fig. 3:**
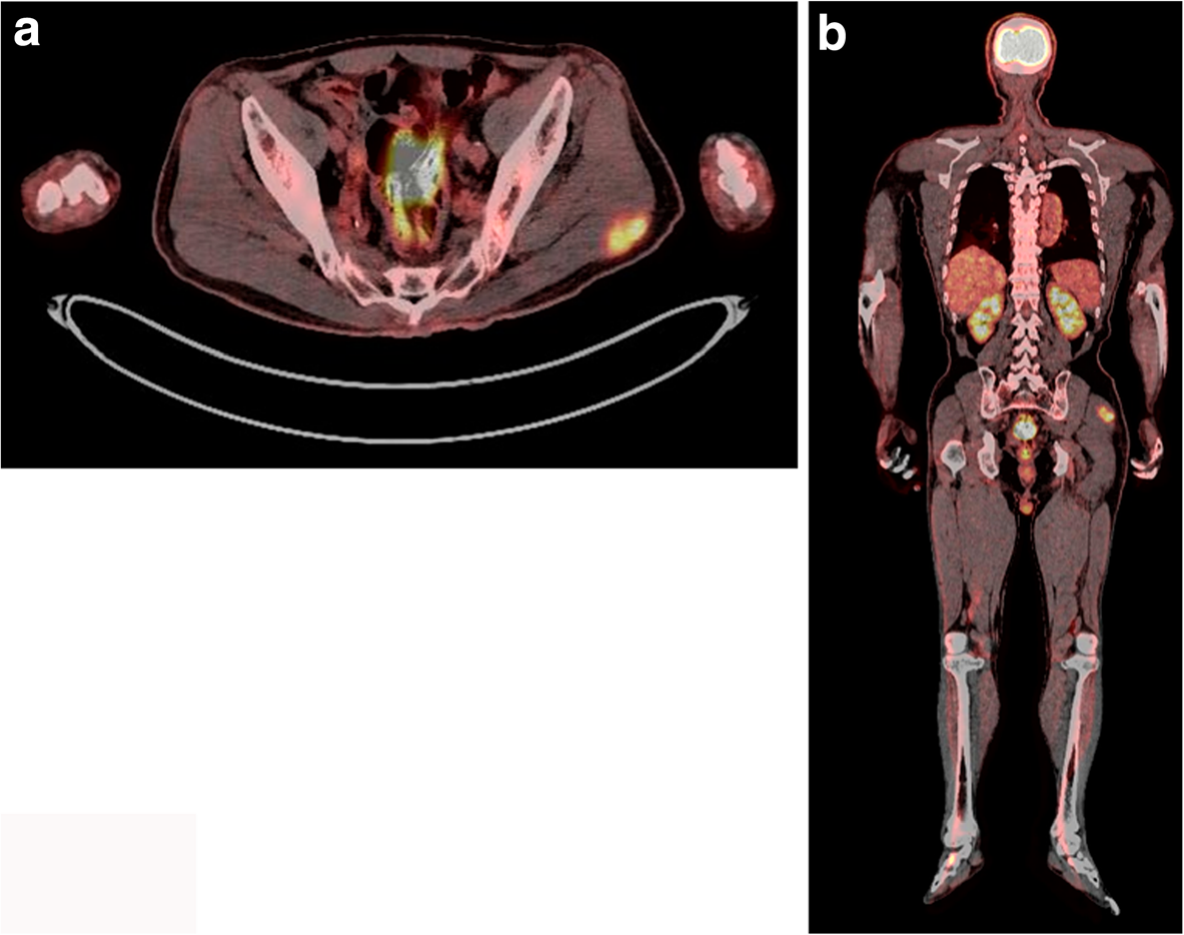
**a**, **b** ^18^F-Fluorodeoxy glucose positron emission tomography revealed positive accumulation of FDG in the left buttock mass (SUV MAX 6.2) but was negative in other locations, including the thyroid gland

A 74-year-old man with no personal or family history of cancer or thyroid disease presented to an orthopedic surgeon with a painful nodule in the left buttock. The nodule was speculated to be an inflammatory soft tissue tumor, and the patient was prescribed anti-inflammatory drugs. However, the mass and symptoms did not improve. He was referred to our hospital because of slow progression in the mass size and pain over a period of 1 year. Physical examination revealed a nodule 30 mm in diameter in the left buttock with tenderness. Laboratory findings were unremarkable, including tumor markers (e.g., carcinoembryonic antigen (CEA), 2.9 ng/ml) and thyroid hormones. Magnetic resonance imaging (MRI) revealed a mass in the left gluteus maximus muscle with T1-weighted image low intensity, T2-weighted image high intensity, and short-TI inversion recovery very high intensity. Enhancement was seen in the mass after administration of gadolinium (Fig. 1a, b). Needle biopsy was performed, and the pathological findings showed adenocarcinoma. The tumor cells were positive for thyroid transcription factor-1 (TTF-1) on immunohistological staining. Computed tomography (CT) of the neck and chest was performed, but there were no abnormal findings, including the thyroid gland (Fig. 2a) and both lungs. Ultrasound examination was also negative for nodules in the thyroid glands (Fig. 2b). Positron emission tomography with fluorodeoxyglucose-computed tomography (FDG-PET/CT) revealed positive accumulation of FDG in the mass in the left buttock (SUVmax 6.2, Fig. 3a) and was negative for other locations (Fig. 3b). Endoscopic examinations, including esophagogastroduodenoscopy and colonoscopy, revealed no abnormal findings. The soft tissue tumor was diagnosed as metastatic adenocarcinoma of unknown primary origin. The tumor was resected with wide margin to control local pain and prevent local progression.

**Fig. 4:**
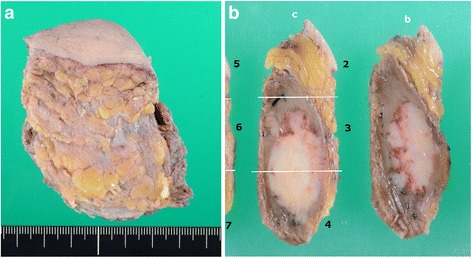
Macroscopic findings of the resected mass (**a** size 80 × 55 × 25 mm). The cut surface revealed a white solid tumor within the skeletal muscle. The size of the tumor was 33 × 18 mm (**b**)

**Fig. 5:**
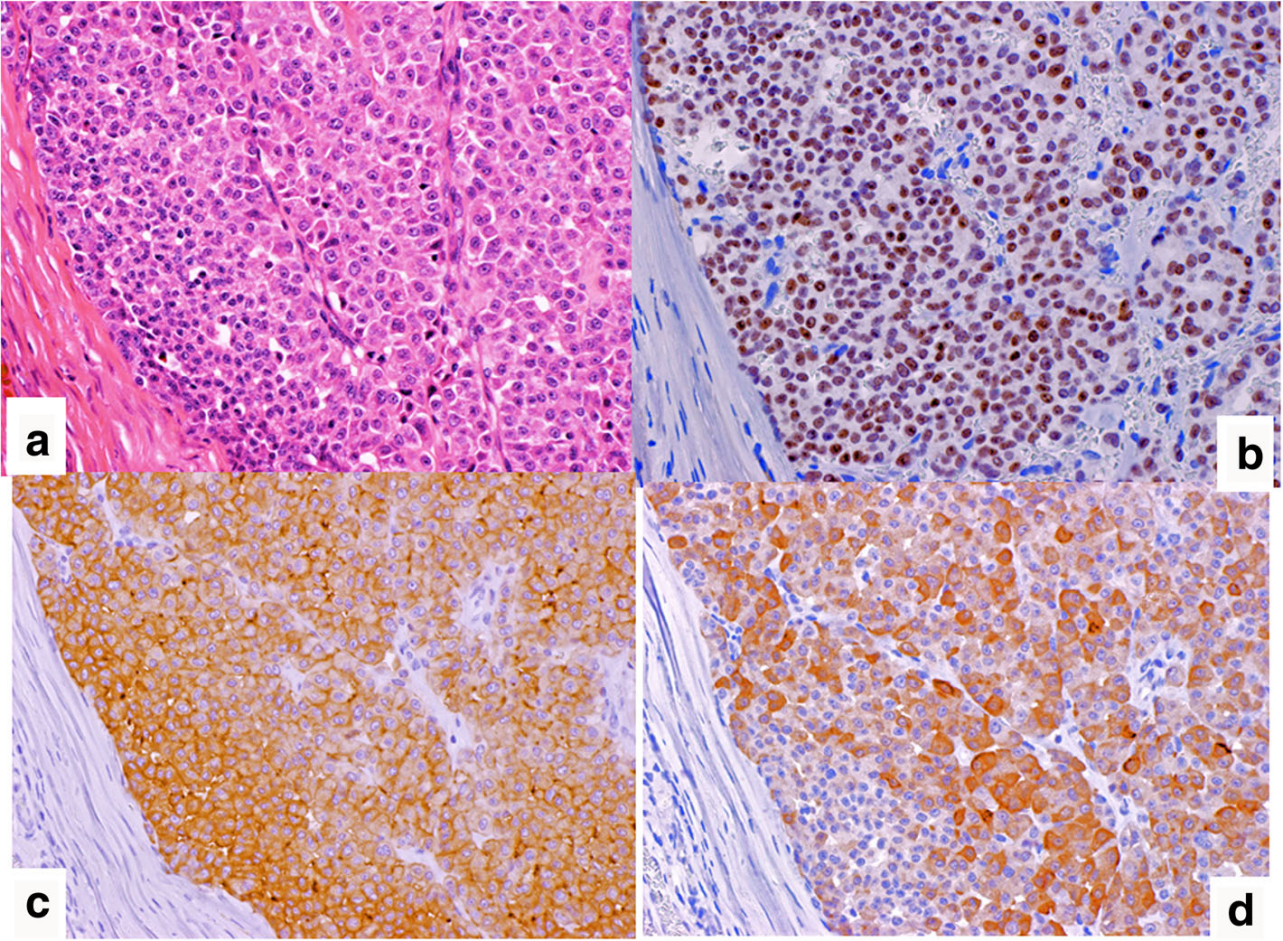
Histological findings revealed the presence of tumor cells, which were polygonal with pale eosinophilic cytoplasm, formed nests, trabeculae, or glands. The nuclei were round to oval in shape with coarse chromatin (**a**). Tumor cells were positive for thyroid transcription factor-1 (TTF-1) (**b**), carcinoembryonic antigen (CEA) (**c**), and calcitonin (**d**) on immunohistological analysis

The resected specimen measured 80 × 55 × 25 mm and weighed 74 g. The cut surface showed a white solid tumor within the skeletal muscle. The size of the tumor was 33 × 18 mm (Fig. 4). Microscopically, tumor cells, which were polygonal with pale eosinophilic cytoplasm, formed nests, trabeculae, or glands. The nuclei were round to oval in shape with coarse chromatin (Fig. 5a). Mitotic activity was low (a single mitosis per 10 HPF). The surgical margin was negative. Immunohistochemically, tumor cells were positive for cytokeratin (CK)-AE1/3, CK7, TTF-1 (Fig. 5b), CEA (Fig. 5c), calcitonin (Fig. 5d), synaptophysin, and chromogranin A, but negative for CK20, Napsin A, Pax8, and p40. These findings indicated metastasis of MTC. Congo red staining was negative for amyloid deposition.

Plasma calcitonin was measured after resection and showed a normal level (4.94 pg/ml). Rearranged during transfection (RET) proto-oncogene mutations were examined but not detected at least in exons 10, 11, and 16. The local pain was relieved after resection of the soft tissue mass, and the patient has remained well without any recurrence or relapse of the disease for over 1 year after resection. In addition, no mass has been undetected in the thyroid gland and plasma calcitonin and CEA levels remain to be within the normal limits.

## Discussion

It has been reported that clinical neck lymph node metastases are detected in at least 50% of patients [1–5] with MTC and that distant metastases are present in 5% [5] or 10–20% [1] of cases of MTC by the time of primary diagnosis. Metastatic sites outside the neck are usually observed in the liver, lungs, or bones.

Single soft tissue metastasis of MTC is extremely rare, although several case series show an unusual presentation of distant metastases of MTC [6–8]. We searched the PubMed database using the keywords “medullary thyroid carcinoma or MTC”, “cutaneous or subcutaneous metastasis,” and “soft tissue metastasis.” We found several cases reports of cutaneous metastasis of MTC [7, 8]. However, to our knowledge, this is the first report of MTC metastatic to soft tissue, and we emphasize that distant metastasis to soft tissue in patients with MTC is extremely rare. Based on a case series study, Glockner et al. [9] reported 11 patients with soft tissue metastases in a group of 1421 patients with a solitary mass over a 14-year period. An autopsy series suggested a higher incidence of metastasis to skeletal muscle [10], but indicated that metastasis to soft tissue cannot be formed without the extensive presence of tumor cells in other organs. Thus, a single soft tissue metastasis of MTC without involvement of other organs presented in our case is a further extremely rare clinical manifestation.

In addition, it is noteworthy that the primary tumor was not detected in the thyroid gland by several imaging examinations since the initial clinical presentation in our case. Identification of cutaneous and/or subcutaneous metastasis is generally a harbinger of widely disseminated disease [7, 8]. Several cases presenting with distant metastasis of occult primary MTC were documented [11–14]. However, these cases had small but identical nodules in the thyroid gland as determined by imaging evaluation, which was quite different from our case. Therefore, we speculated that this metastatic MTC was unknown primary origin or that primary lesion in thyroid glands showed spontaneous regression to undetectable size. It has been shown that long survival times have been observed without any systemic treatment in a few patients with metastatic MTC, especially in initially disseminated diseases [4, 5]. Indeed, the soft tissue mass in the left buttock of our patient grew slowly over 1 year and the tumor cells showed a low mitotic index. Based on these clinical findings, the primary tumor may develop in future in the present case. Careful and long-term follow-up are needed.

It is well known that analysis of the CK7/CK20 immuno-phenotypes and/or tissue-specific antibody is useful for determination of the primary site in metastatic adenocarcinoma [15]. Yoshimura et al. [16] described the usefulness in soft tissue metastasis not only to determine the primary tumor site correctly but also to differentiate the soft tissue metastasis of carcinoma from soft tissue sarcoma. In the present case, TTF-1, a tissue-specific antibody for the thyroid and lung, was essential for the exact diagnosis. Thus, we emphasize the importance of detailed clinical observation and immunohistochemical analyses for metastatic tumors.

## Conclusions

In conclusion, we described a case of single subcutaneous metastasis of MTC without a preexisting thyroid mass. Our experience indicated that the disease could exhibit a variety of clinical manifestations and detailed immunohistochemical examinations are important for primary unknown origin.

## Abbreviations

FDG-PET: Positron emission tomography with fluorodeoxyglucose
MTC: Medullary thyroid carcinoma
RET: Rearranged during transfection.
TTF-1: Thyroid transcription factor-1
